# Uroperitoneum as a Complication of Laparoscopic Cholecystectomy: A Case Report

**DOI:** 10.3390/jpm13040696

**Published:** 2023-04-21

**Authors:** Charalampos Kefalas, Alexandra Menni, Eleni Karlafti, Stavros Panidis, Georgios Chatziantoniou, Konstantina Katsiafliaka, Despina Krokou, Aristeidis Ioannidis, Patroklos Goulas, Smaro Netta, Daniel Paramythiotis

**Affiliations:** 1 1st Propaedeutic Surgery Department, AHEPA University General Hospital of Thessaloniki, Aristotle University of Thessaloniki, 54636 Thessaloniki, Greece; alexandra061208@gmail.com (A.M.); st.panidis@gmail.com (S.P.); georchatziantoniou@gmail.com (G.C.); konstantina.katsi@gmail.com (K.K.); despik@windowslive.com (D.K.); ariioann@yahoo.gr (A.I.); patroklos@live.com (P.G.); smaronetta2@gmail.com (S.N.);; 2 Emergency Department, AHEPA University General Hospital of Thessaloniki, Aristotle University of Thessaloniki, 54636 Thessaloniki, Greece; linakarlafti@hotmail.com

**Keywords:** uroperitoneum, bladder rupture, laparoscopic cholecystectomy, pseudorenal failure, iatrogenic injury

## Abstract

Background: Bladder rupture is more frequently encountered in blunt pelvic trauma, but can also be spontaneous or iatrogenic. Laparoscopic repair has been widely used during the last few years as a treatment for intraperitoneal bladder perforation. The bladder is the genitourinary organ most often affected by iatrogenic injury. The purpose of this article is to report what is, to our knowledge, the first documented case of bladder rupture as a complication of laparoscopic cholecystectomy. Case description: A 51-year-old female presented to the emergency department complaining about generalized abdominal pain on the sixth postoperative day after laparoscopic cholecystectomy. Laboratory results highlighted a significant impact on renal function while the abdominal CT scan demonstrated free intraperitoneal fluid collection and surgical clips in the anatomic region of the liver and in an ectopic position near the ileocecal valve. An explorative laparoscopy revealed a 2 cm defect in the superior bladder wall, which was repaired in one layer in a continuous-locking fashion. The patient was discharged home on the fifth postoperative day having an uneventful recovery. Conclusion: Bladder rupture frequently presents with non-specific clinical signs; as a result, it is easily misdiagnosed, especially when it occurs with a non-typical mechanism of injury. Pseudorenal failure is a relatively obscure entity that may help the clinician suspect a bladder perforation. Laparoscopic repair with a single-layer continuous suture technique is a safe and feasible treatment in hemodynamically stable patients. Prospective research is required to specify the optimal timing of catheter removal after bladder repair.

## 1. Introduction

Bladder rupture, although a rare entity, is more frequently encountered in blunt pelvic trauma, but can also be spontaneous or iatrogenic [1]. Bladder perforations are classified as extraperitoneal (50–71%), intraperitoneal (25–43%), or combined (7–14%) [2]. Most extraperitoneal bladder ruptures (EBRs) can be managed conservatively with bladder drainage, supportive measures, and follow-up cystography [3]. Some authors have cited several factors that preclude the use of conservative management in certain cases, such as: the presence of a bone fragment that protrudes into the rupture making it impossible to heal, rectal perforation, and open pelvic fracture. However, cases involving these bone fragments are rare. Additionally, open pelvic fractures and rectal perforations pose a significant risk of severe infection if treated with conservative management [4]. On the contrary, intraperitoneal bladder ruptures (IBRs) are conventionally treated with laparotomy and bladder suturing [2]. Laparoscopic repair is also an option, and has been widely used during the last few years, as it is associated with less postoperative pain, decreased hospitalization, and earlier return to work [5].

The typical clinical presentation of a patient with bladder injury is suprapubic tenderness, gross hematuria, and/or blood per urethra [6]. Moreover, IBR should be considered in the differential diagnosis of a trauma patient or a patient with a history of recent abdominal surgery or transurethral procedure complaining about abdominal pain and inability to void [7].

Although iatrogenic bladder rupture is less common than traumatic, the bladder is the genitourinary organ most often affected by iatrogenic injury [7]. Iatrogenic bladder injuries can be classified as those ascribed to internal bladder procedures and those occurring during intra-abdominal operations. Internal injuries more frequently occur during transurethral bladder tumor resection [8]. A wide variety of intra-abdominal operations can also lead to bladder injury with hysterectomy, cesarian section, and pelvic mass resection being most common. Regarding general surgery operations, colon resection due to malignancy is the most common procedure causing bladder injury [9]. To our knowledge, this is the first reported case of bladder perforation during laparoscopic cholecystectomy.

The most common complications following laparoscopic cholecystectomy are bile duct, bowel or vascular injuries, and postoperative bile leak or hemorrhage. Bile duct injury (excluding the cystic duct) has an average rate of occurrence of 0.6%. However, the most dangerous complications are bowel and vascular injuries, which occur in 0.14% and 0.25% of cases, respectively. Postoperative bile leakage is identified in 0.3% of patients, with the most frequent source being the cystic duct [10].

## 2. Case Description

**Figure 1:**
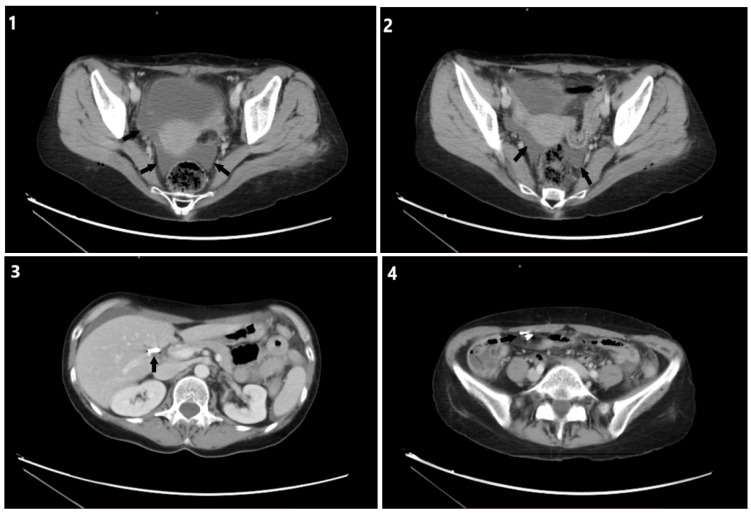
CT abdomen; (**1**,**2**) Free intraperitoneal fluid with similar density to urine in the bladder (black arrows) (**3**) Surgical clips in the anatomic position of the gall bladder (black arrow). (**4**) Surgical clips in an ectopic position near the ileocecal valve (black arrow).

A 51-year-old female presented to the emergency department complaining about generalized abdominal pain on the sixth postoperative day after laparoscopic cholecystectomy. The patient had a clear further medical history, was not taking any medication, and did not have any allergies. Moreover, she reported smoking 1 pack/per day and no alcohol consumption. Vital signs were 101 beats/min, blood pressure 110/60 mmHg, respiration 18 breaths/min, and body temperature 37 °C. Physical evaluation of the patient revealed severe diffuse abdominal pain, muscular defense, and rebound tenderness periumbilicus and in the hypogastrium area. Laboratory results at the time of admission did not reveal markers of inflammation, but highlighted a significant impact on renal function (Table 1). An abdominal CT scan revealed free intraperitoneal fluid collection and surgical clips in the anatomic region of the liver and in an ectopic position near the ileocecal valve [Figure 1]. A diagnostic laparoscopy was decided to evaluate the exact cause of the free fluid with a high suspicion of chyloperitoneum existence.

**Figure 2:**
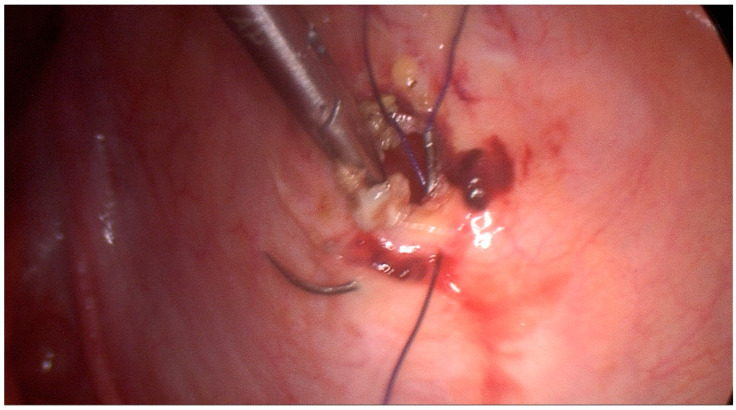
Intraoperative view and suturing of the bladder perforation.

**Figure 3:**
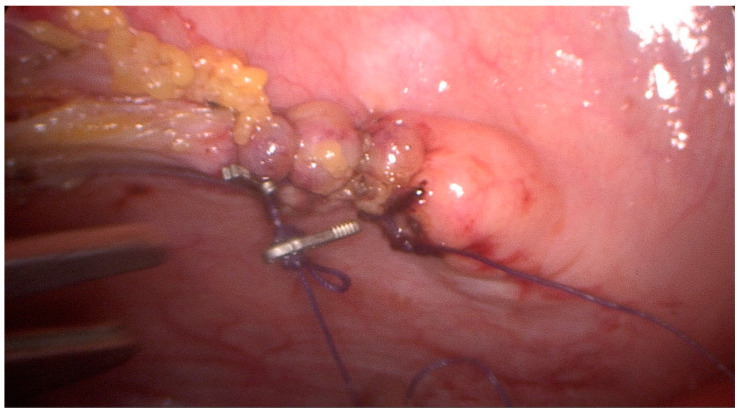
Single-layer continuous-locking repair of the bladder deficit with vicryl 1–0 and surgical clips for stabilization of the knots.

Particularly, with the patient under general anesthesia, in a supine position, the pneumoperitoneum was obtained using Hasson’s technique via a sub-umbilical curvilinear incision and insertion of an 11 mm Hasson trocar. Another two 5 mm trocars were inserted under direct vision in the previous sites of the laparoscopic cholecystectomy (right midclavicular line and right anterior axillary line). Inspection of the peritoneal cavity revealed a collection of serous fluid in the lesser pelvis while the surgical clips of the laparoscopic cholecystectomy were on site with no sign of bile leak. A more detailed inspection of the lesser pelvis revealed a 2 cm defect in the superior bladder wall [Figure 2]. The bladder rupture was repaired in one layer using a 1–0 vicryl suture in a continuous-locking fashion [Figure 3]. Afterward, the bladder was filled with saline through a Foley catheter, confirming the adequacy of the repair. Subsequently, the abdominal cavity was irrigated with sterile water and a 20 mm drain was placed in the vesicouterine pouch. The drain was removed on the second postoperative day after no signs of urine extravasation in the abdominal cavity. The patient was discharged home on the fifth postoperative day on oral antibiotics having an uneventful recovery. The urinary catheter was removed 21 days later. Two months after the operation, follow-up revealed an uneventful recovery with no complications.

## 3. Discussion

**Figure 4:**
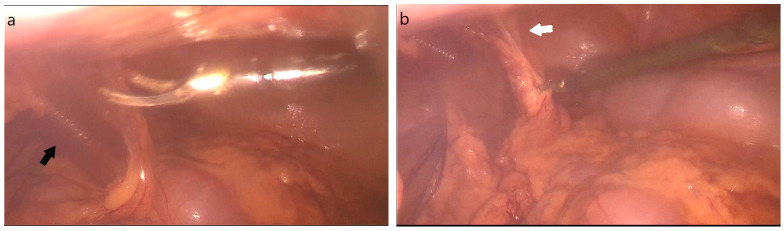
Adhesiolysis (white arrow in (**b**) showing adhesions) near the sub-umbilical trocar (black arrow in (**a**)), that possibly led to bladder perforation either by mechanical trauma or via an electrothermal injury. The bladder injury was not noticed intra-operatively.

Urinary bladder perforations are more often encountered in polytrauma patients with blunt pelvic injury. However, they can also be iatrogenic or spontaneous [1]. The occurrence of spontaneous bladder perforations (SBP) without any associated trauma is an infrequent phenomenon, with a documented occurrence of approximately 1 in 126,000 cases. The two leading factors contributing to SBP are alcohol intoxication, accounting for 39.2% of cases, and lower urinary tract obstruction, which accounts for 18.3% of cases [7]. A grading system to classify the intensity of bladder injuries has been proposed by the American Association of Surgical Trauma, although ruptures are more practically categorized as extraperitoneal, intraperitoneal, or combined [11]. Iatrogenic bladder injuries are more frequently ascribed to urological, gynecological, and general surgery operations. Hysterectomy and transurethral resection of bladder tumors are the most common procedures resulting in bladder rupture [8]. Fortunately, most iatrogenic bladder perforations are recognized and managed intra-operatively, as delay in diagnosis can result in urinary fistula, organ loss, and sepsis [12]. When direct visualization of the injury is not possible, bladder perforation may be suspected by visualization of urine on the operative field or air in the urine collection bag [8,9]. In our case, the bladder deficit was not noticed intra-operatively and the patient presented in our emergency department with acute abdomen due to uroperitoneum. The patient provided us with a video recording of the initial laparoscopic cholecystectomy, wherein the operating surgeon appeared to perform adhesiolysis below the level of the sub-umbilical trocar [Figure 4]. The adhesions appear in the anatomical place of the urachus, which is directly connected with the urinary bladder. Considering that the patient did not void prior to the operation and a Foley catheter was not placed, the bladder perforation could have occurred either by mechanical trauma or via an electrothermal injury with the diathermy used for adhesiolysis.

The clinical signs that are often present in bladder rupture are, in the majority of cases, non-specific. Nonetheless, gross hematuria, suprapubic pain or tenderness, and difficulty or inability to void are a triad of symptoms that are frequently noticed [13]. CT cystography is the gold standard for the diagnosis of bladder perforation [8]. In the context of EBRs, retrograde CT cystography reveals the extravasation of contrast material into the pelvic region. In contrast, when dealing with IBRs, contrast leaks into the peritoneal cavity filling intra-abdominal spaces and/or outlining bowel loops. To ensure accurate assessment, adequate bladder distension with the infusion of a minimum of 350 mL of contrast is necessary, and imaging after bladder drainage should also be included. This method has been reported to have an accuracy ranging from 85% to 100%. On the other hand, flawed imaging techniques, such as the use of only 250 mL of contrast for bladder infusion or failure to include post-bladder drainage imaging, may lead to a significant number of false negative results [8]. However, in our case, bladder perforation was not suspected at the patient’s admission due to the non-typical mechanism of injury, since bladder rupture is not a known complication of laparoscopic cholecystectomy. Thus, a conventional abdominal CT scan was performed. As in our case, when an IBR is not treated early, urine leaks into the peritoneal cavity resulting in a uroperitoneum. This occurs as with continuous urine extravasation; the patient is unable to remove the excess peritoneal fluid due to the fact that the excretion of kidney function significantly transcends subdiaphragmatic lymph flow [14]. Urine consists of a higher level of creatinine and nitrogen waste products as compared with serum [15]. As a result, the peritoneum serves as a semipermeable membrane allowing concentration gradient diffusion while urine is in contact with the peritoneum, a condition termed reversed auto-dialysis [16]. As demonstrated in our case, the serum creatinine level rises while the glomerular filtration rate of the patient is practically stable [15]. This condition, although relatively obscure to most physicians, is referred to as pseudorenal failure and should be in the differential diagnosis of a patient with recent abdominal trauma or surgery that presents with abdominal distention and elevated serum creatinine level, with no history of kidney dysfunction [17].

Even though EBR is typically managed conservatively with bladder catheterization for drainage and supportive care, IBR principally requires laparotomy and bladder perforation suturing [3]. When hemodynamic instability is present, exploratory laparotomy remains the golden standard [18]. Nevertheless, in patients that remain hemodynamically stable, laparoscopic exploration of the abdominal cavity for other injuries and closing the deficit with laparoscopic suturing seems to be a safe and sufficient option [18]. The laparoscopic approach has proven to be equivalent to the standard laparotomy technique concerning the evaluation of bowel viability and other abdominal injuries into the peritoneal cavity [19]. Furthermore, it has the benefit of earlier return to work, less postoperative pain, and decreased hospitalization, with the only disadvantage being the longer operative time that is frequently needed [5,12]. Regarding iatrogenic bladder perforations, such as in our case, usually there is a single bladder defect in the posterior bladder wall or near the dome, areas accessible via a laparoscopic approach [20]. However, if the deficit is near the trigone or close to the ureter, a more conventional approach with a standard laparotomy may be the only option [18,20]. A bladder perforation is traditionally repaired in two layers [3,8]. Nevertheless, it has been shown in several studies that a single-layer continuous locking repair is sufficient and there is no difference in the outcome compared to the conventional two-layer approach [2,3,19]. 

The placement of a suprapubic catheter is not supported by evidence either alone or combined with a urethral catheter, but it may be helpful in selected cases when prolonged catheterization is anticipated or when there is tension in the bladder closure [2,3]. Transurethral Foley catheterization alone is an adequate method after surgical repair of a bladder perforation [3]. The adequacy of the bladder repair can be assessed with the insertion of normal saline or methylene blue through the Foley catheter during the operation [2]. Conventionally, cystography is performed 7–10 days after surgical repair of the bladder to evaluate the efficacy of the repair [4]. In this case, the patient did not attend the follow-up cystography and the catheter was removed on the 21st postoperative day. There seems to be no benefit in postoperative cystography more than 7–10 days after the operation; hence, the catheter was removed without it being performed [21]. Moreover, there is not enough evidence in the international literature regarding the optimal timing of catheter removal and further prospective evaluation is required [21].

## 4. Conclusions

In summary, although rare, bladder rupture is a complication that may be encountered after laparoscopic cholecystectomy. As the associated clinical signs are frequently non-specific, it is a condition easily that is misdiagnosed. Pseudorenal failure is a relatively obscure entity that may help the clinician suspect bladder perforation. Furthermore, laparoscopic repair of a bladder rupture in hemodynamically stable patients is a safe and sufficient procedure with additional benefits compared to laparotomy. As a final point, more prospective research is required to specify the optimal timing of catheter removal after bladder repair.

## Figures and Tables

**Table 1:** Patient’s laboratory results.

Test	Value	Normal Values
*Complete Blood Count*		
Hematocrit	31.4%	37–47%
Hemoglobin	10.8 g/dl	12–16 g/dl
Red Blood Cell Count	3.65 M/mL	3.8–5.3 M/mL
Mean Corpuscular Volume (MCV)	86 fl	80–99 fl
Mean Corpuscular Hemoglobin (MCH)	29.6 pg	27–32 pg
Mean Corpuscular Hemoglobin Concentration (MCHC)	34.4 g/dl	32–35 g/dl
Red Cell Distribution Width (RDW)	38.2 fl	37–47 fl
White Blood Cell Count	8.66 K/μL	3.8–10.5 K/μL
Neutrophils	85.1%	45–75%
Platelet Count	193 K/μL	150–450 K/μL
*Biochemical Tests*		
Serum Glucose	72 mg/dl	74–100 mg/dl
Serum Urea	63 mg/dl	17–43 mg/dl
Serum Creatinine	4.18 mg/dl	0.66–1.9 mg/dl
Aspartate Transaminase (AST)	12 U/L	<35 U/L
Alanine Transaminase (ALT)	11 U/L	<35 U/L
Gamma-glutamyl Transferase	44 U/L	<38 U/L
C Reactive Protein (CRP)	7.94	<0.5 mg/dl
Serum Potassium	4.2 mmol/L	3.5–5.1 mmol/L
Serum Sodium	143 mmol/L	136–146 mmol/L

## Data Availability

The data presented in this study are available on reasonable request from the corresponding author.
